# Spatial-sweep steady-state pattern electroretinography can detect subtle differences in visual function among healthy adults

**DOI:** 10.1038/s41598-019-54606-z

**Published:** 2019-12-02

**Authors:** Sakiko Minami, Norihiro Nagai, Misa Suzuki, Toshihide Kurihara, Hideki Sonobe, Kazuhiro Watanabe, Hajime Shinoda, Hitoshi Takagi, Kazuo Tsubota, Yoko Ozawa

**Affiliations:** 1 Department of Ophthalmology, Keio University School of Medicine, Tokyo, Japan; 2 Department of Ophthalmology, Inagi Municipal Hospital, Tokyo, Japan; 3 Laboratory of Retinal Cell Biology, Department of Ophthalmology, Keio University, School of Medicine, Tokyo, Japan; 4 Department of Ophthalmology, Yokohama City University, Yokohama, Japan; 5 Department of Ophthalmology, St. Marianna University School of Medicine, Kawasaki, Japan

**Keywords:** Electronic devices, Materials for optics

## Abstract

We aimed to establish a highly sensitive method for measuring visual function using spatial-sweep steady-state pattern electroretinography (swpPERG). Overall, 35 eyes of 35 healthy adults (18 men; mean age, 32.3 years) were examined using swpPERG, and the data were recorded using spatial-patterned and contrast-reversed stimuli of size 1 (thickest) to 6. Data were converted into frequency-domain using a Fourier transform and expressed by signal-to-noise ratio (SNR). The number of participants who showed SNR ≥ 1 was significantly lesser at stimulus sizes 5 and 6 compared with those at greater stimulus sizes. Among the data with SNR ≥ 1, SNRs were negatively correlated with age at stimulus size 5 (r = −0.500, *P* = 0.029), and positively correlated with macular volume evaluated by optical coherence tomography (OCT) within a 6-mm circle diameter from the fovea of the retinal nerve fibre layer at size 4 (r = 0.409, *P* = 0.025) and of the ganglion cell layer at size 5 (r = 0.567, *P* = 0.011). We found that SNRs of swpPERG, recorded using the EvokeDx system, were correlated with age and macular morphology in participants without diagnosed eye diseases. The system detected subtle differences in retinal function, which may help in early disease diagnosis and visual evaluation in neuroprotective interventions in the future.

## Introduction

The increase in the prevalence of age-related eye diseases is an issue that warrants attention in the current aging society^1^. Because many diseases leading to blindness, such as those affecting the retina and glaucoma^2^, are irreversible once progressed, early diagnosis^3,4^ and preventive interventions^5^ are crucial for maintaining patients’ quality of life. Thus, detecting subtle differences in visual function has clinical value in diagnosis and treatment planning. Moreover, clinical trials for neuroprotective therapies are now on-going worldwide; these require the ability to assess and confirm small differences in visual function. Most neurodegenerative diseases progress gradually and currently have no effective treatments; thus, identifying very small changes in visual function during clinical trial is indispensable. However, commonly used current methods to measure visual function such as the Snellen and Landolt’s charts may not detect subtle changes^6,7^. Thus, sensitive and, in particular, objective methods to detect these subtle changes in visual function are essential.

We have previously reported subjective visual measurement methods with the ability to detect small changes. Functional visual acuity and contrast visual acuity measurement methods quantified differences in function among patients with visual acuity better than 0 in LogMAR (20/20 in the Snellen chart, and 1.0 in the Landolt’s chart), while they already had idiopathic epiretinal membrane^6^ or age-related macular degeneration (AMD)^7^. These methods may be used to detect subtle changes subjectively among patients who have been diagnosed with minor illness. However, these methods confirmed visual function changes among patients who had already noticed their symptoms of blurred and/or distorted vision. The concept of detecting preperimetric glaucoma by optical coherence tomography (OCT) imaging in patients without a glaucomatous visual field defect as well as symptoms^8^ has recently attracted attention. A paradigm shift towards early diagnosis for preventive therapy requires sensitive and objective methods that can detect visual changes among people without symptoms or eye disease diagnosed by current methods. Moreover, clinical studies on interventions to preserve or improve the retinal neural condition by neuroprotective drugs^9^, gene therapies, and stem cell therapies^10^ for retinal degeneration such as dry AMD and hereditary retinal degenerations may require the detection of subtle differences in visual function.

Here, we report the usage of spatial-sweep steady-state pattern electroretinography (swpPERG) to detect subtle changes and variations of visual function among healthy adults with no diagnosed eye diseases. The system presents the spatial pattern (horizontal grating) images with contrast-reverses in time at 7.5 Hz (15 reversals per second) to record the ERG responses. This stimulus maintains a constant space-average luminance so that the scattered light produced in the eyeball by the optical media does not change over time, and, therefore, does not elicit a response from the retina beyond the area directly stimulated by the patterned field. The averaged complex waveform is converted to the frequency-domain using a Fourier transform to deconstruct the complex waveform into its frequency components to identify objective measures that capture the entire set of information in the response for analysis, instead of only a few time points^11–13^. Then, responses corresponding to the 10 Hz stimulus are expressed as signal-to-noise ratio (SNR). Thus, the influences of noise (produced by eye movements and other electromagnetic fields in the testing environment) can be excluded from the data for analysis^14^. A similar system using the spatial sweep in the visual evoked potential technique was first described by Zemon *et al*.^14^. However, the usage of swpPERG is scarcely reported. Thus, the current study aimed to use swpPERG to identify these subtle changes in visual function.

We believe that quantifying changes in visual function via swpPERG in people without symptoms or diagnosed disease could aid in early diagnosis of retinal conditions and evaluating treatment effects in the clinic. It will also help aid future clinical studies that require quantification in visual function such as studies on early diagnosis and neuroprotection.

## Results

**Figure 1:**
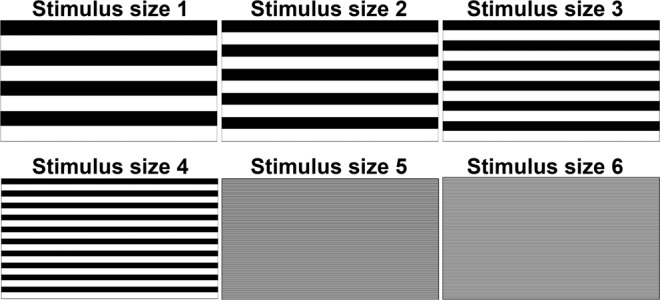
Stimulus sizes 1–6. The figure depicts the stimulus size, where 1 is the thickest and 6 is the thinnest.

**Figure 2:**
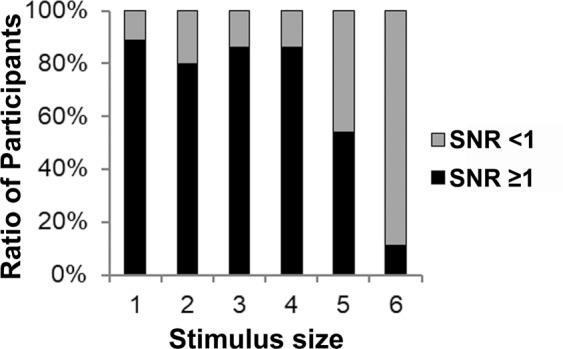
Ratio of participants with SNR ≥ 1 at the respective stimulus sizes. SNR, signal to noise ratio.

Among 35 adults, 18 (51%) were men (Table 1). The mean age was 32.3 ± 7.6 years (range 22–48), the best-corrected visual acuity (BCVA) was −0.079 in LogMAR among all participants, and the mean refractive error was −2.3 ± 2.1D (range −5.9 to + 1.4D). The mean SNR at stimulus size 1 (thickest), 2, 3, 4, 5, and 6 (Fig. 1) was 1.63 ± 0.68, 1.80 ± 0.84, 1.82 ± 0.86, 1.83 ± 0.91, 1.27 ± 0.72, 0.71 ± 0.48, respectively (Table 1); the mean SNR at stimulus size 6 was lower than that at stimulus size 1 (*P* < 0.001). The degree of visual function was defined as follows: the value of SNR represented the ability of visual function when SNR ≥ 1, and the data with SNR < 1 shows that signals from the eye were smaller than noise, and the signal was considered undetected. SNR ≥ 1 was observed in 31 eyes (89%) at stimulus size 1, 28 eyes (80%) at stimulus size 2, 30 eyes (86%) at stimulus size 3, 30 eyes (86%) at stimulus size 4, 19 eyes (54%) at stimulus size 5, and 4 eyes (11%) at stimulus size 6 (Fig. 2, Table 2, *P* values are shown in the table). The number of participants who showed SNR ≥ 1 was significantly lesser at stimulus sizes 5 and 6 compared with those at greater stimulus sizes (Table 2). Furthermore, participants showed SNR ≥ 1 less frequently at stimulus size 6 than that at stimulus size 5 (Table 2, *P* < 0.001). The results suggested that the response was masked by the noise, and not detected in more participants at thinner stimulus sizes. This is most likely because thinner stimuli were difficult for some participants to respond to.

**Table 1:** Characteristics of the Participants (n = 35)

		(Mean ± SD)
Age (yrs)	22 to 48	(32.3 ± 7.6)
Gender (male [%])	18 (51)	—
BCVA (LogMAR)	all −0.079	(−0.079 ± 0.00)
Refraction (Diopters)	−5.9 to +1.4	(−2.3 ± 2.1)
SNR		
Stimulus size 1	0.75 to 3.65	(1.63 ± 0.68)
Stimulus size 2	0.48 to 3.58	(1.80 ± 0.84)
Stimulus size 3	0.32 to 3.59	(1.82 ± 0.86)
Stimulus size 4	0.57 to 4.54	(1.83 ± 0.91)
Stimulus size 5	0.35 to 3.16	(1.27 ± 0.72)
Stimulus size 6	0.16 to 2.84	(0.71 ± 0.48)

BCVA, best-corrected visual acuity; SNR, signal to noise ratio.

**Table 2:** Number of Participants who Showed SNR ≥ 1 at Respective Stimulus Sizes and the Differences of the Numbers between the Stimulus Sizes

SNR ≥ 1				*P*
Stimulus size	n (%)	Stimulus size	n (%)	
1	31 (89)	**2**	28 (80)	0.505
		**3**	30 (86)	1
		**4**	30 (86)	1
		**5**	19 (54)	0.003**
		**6**	4 (11)	<0.001**
2	28 (80)	**3**	30 (86)	0.724
		**4**	30 (86)	0.724
		**5**	19 (54)	0.027*
		**6**	4 (11)	<0.001**
3	30 (86)	**4**	30 (86)	1
		**5**	19 (54)	0.006**
		**6**	4 (11)	<0.001**
4	30 (86)	**5**	19 (54)	0.009**
		**6**	4 (11)	<0.001**
5	19 (54)	**6**	4 (11)	<0.001**

McNemar test. P values, comparisons with the value of stimulus size 1. SNR, signal to noise ratio. *P < 0.05, **P < 0.01.

**Figure 3:**
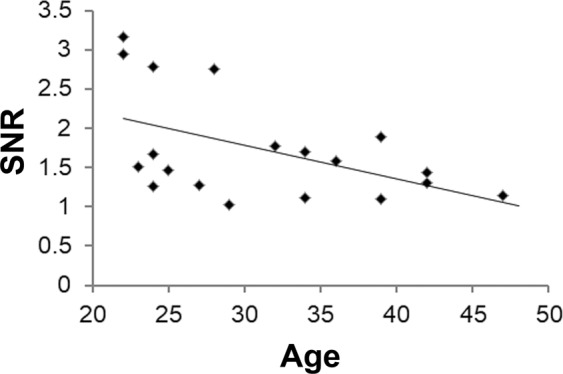
Negative correlation between SNR and age at stimulus size 5 among participants with SNR ≥ 1. Analyses performed by Pearson correlation coefficient. r = −0.500, *P* = 0.029. SNR, signal to noise ratio.

Participants with SNR < 1 were significantly older than those with SNR ≥ 1 at respective stimulus sizes of 1 and 4 (Table 3, *P* = 0.012, and 0.049, respectively). Other stimulus sizes showed a similar trend. In addition, there was a significant negative correlation between SNR and age at stimulus size 5 among participants with SNR ≥ 1 (Fig. 3, r = −0.500, *P* = 0.029). At stimulus sizes 3 (r = −0.308, *P* = 0.097) and 4 (r = −0.312, *P* = 0.093), there were trends of negative correlations between SNR and age (Supplemental Table 1). Taken together, older participants showed smaller visual signals, and resulted in obtaining SNR < 1 more frequently. There were no differences in the SNR between males and females, and no relationships between SNR and refractions (data not shown).

**Table 3:** Comparison of Age in Participants Who Showed SNR ≥ 1 and <1 at Respective Stimulus Size.

	SNR ≥ 1	SNR < 1	*P*
Stimulus size 1	31.16 ± 7.06	41.00 ± 5.94	0.012*
Stimulus size 2	32.14 ± 6.70	32.86 ± 10.16	0.827
Stimulus size 3	31.50 ± 7.13	37.00 ± 9.22	0.134
Stimulus size 4	31.27 ± 6.95	38.40 ± 9.02	0.049*
Stimulus size 5	31.21 ± 7.84	33.56 ± 7.25	0.367
Stimulus size 6	26.75 ± 4.86	33.00 ± 7.60	0.121

Two-tailed t-test. SNR, signal to noise ratio. *P** < **0.05.

**Figure 4:**
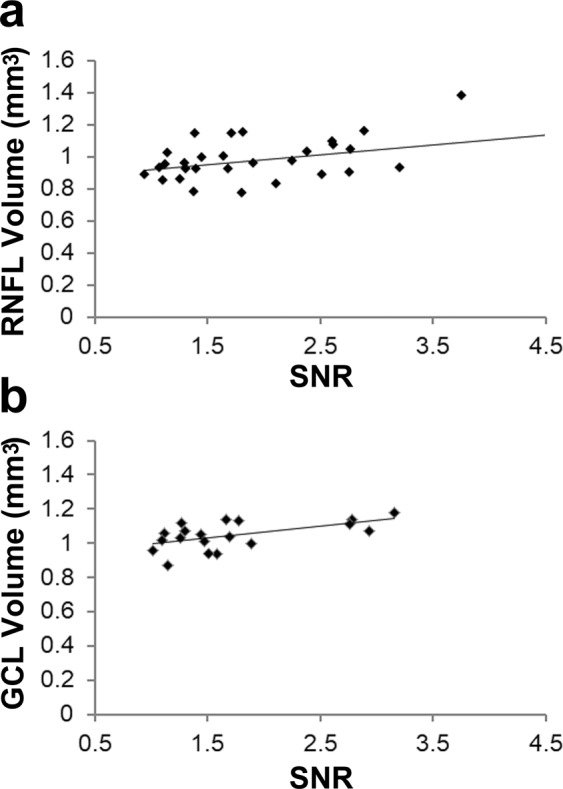
Positive correlations between SNR and macular volumes. (**a**) Retinal nerve fibre layer (RNFL) and (**b**) ganglion cell layer (GCL) volumes were measured by three-dimensional optical coherence tomography (OCT). Analyses performed by Pearson correlation coefficient. (**a**) r = 0.409, *P* = 0.025; (**b**) r = 0.567, *P* = 0.011. SNR, signal to noise ratio.

We then analysed the correlations between SNR and retinal neuronal volume as evaluated by 3 dimensional OCT images using the data of participants whose SNR was over 1 (Supplemental Table 2). We found a positive correlation between SNR and retinal nerve fibre layer (RNFL) volume at stimulus size 4 (Supplemental Table 2, Fig. 4a, r = 0.409, *P* = 0.025) and SNR and ganglion cell layer (GCL) volume at stimulus size 5 (Supplemental Table 3, Fig. 4b, r = 0.567, *P* = 0.011). Moreover, the RNFL volume of the participants who had SNR ≥ 1 at stimulus size 6 was significantly greater than hat who had SNR < 1 (Table 4).

**Table 4:** Comparison of Retinal Nerve Fibre Layer and Ganglion Cell Layer Volumes between Participants Who Showed SNR ≥ 1 and <1 at Respective Stimulus Size.

RNFL Volume	SNR ≥ 1	SNR < 1	*P*
Stimulus size 1	0.991 ± 0.135	0.953 ± 0.036	0.579
Stimulus size 2	1.003 ± 0.126	0.923 ± 0.125	0.143
Stimulus size 3	0.980 ± 0.131	1.028 ± 0.108	0.443
Stimulus size 4	0.981 ± 0.129	1.020 ± 0.134	0.536
Stimulus size 5	0.976 ± 0.146	0.999 ± 0.106	0.613
Stimulus size 6	1.123 ± 0.177	0.969 ± 0.113	0.022*
**GCL Volume**			
Stimulus size 1	1.044 ± 0.017	1.075 ± 0.051	0.529
Stimulus size 2	1.048 ± 0.089	1.046 ± 0.105	0.949
Stimulus size 3	1.036 ± 0.091	1.120 ± 0.047	0.052
Stimulus size 4	1.050 ± 0.083	1.036 ± 0.137	0.759
Stimulus size 5	1.046 ± 0.081	1.049 ± 0.103	0.922
Stimulus size 6	1.093 ± 0.106	1.042 ± 0.089	0.299

Two-tailed t-test. RNFL, retinal nerve fibre layer; GCL, ganglion cell layer; SNR, signal to noise ratio. *P < 0.05.

## Discussion

In this study, we demonstrated that visual function measured by swpPERG using the EvokeDx^®^ system showed variations of SNR among healthy adults, in particular, in those with smaller stimulus sizes. We found a negative correlation between SNR and age, and a positive correlation between SNR and RNFL, and SNR and GCL volumes as measured by OCT.

An SNR ≥ 1 corresponds to visual function. however, SNR < 1 indicates that noise is exceeding the visual response; thus, visual response is undetectable. Data from participants with “undetectable” visual responses was obtained, indicating that, even in the healthy adults, there were variations or subtle differences in the visual function among individuals as confirmed by using the EvokeDx^®^ system. Moreover, undetectable results were obtained at smaller stimulus sizes, which is a plausible result.

The mean age was significantly older in participants with SNR < 1, where in the visual response was undetectable. In addition, among the participants who showed a detectable response, there was a significant negative correlation between SNR and age at stimulus size 5. These results suggested that age-related reduction in visual responses among those without diagnosed diseases can be detected by swpPERG measurements using the EvokeDx^®^ system. In particular, stimulus size 5 was thin and sensitive enough to detect the differences. In contrast, most participants well responded to thicker stimuli irrespective of age, and stimulus size 6 was too thin and many of the participants did not respond to, thus, no differences were shown between individuals.

An age-related reduction in visual response was reported in adults over 50 years of age using the ordinary scotopic ERG^15^. In the current study, age-related visual changes were observed among the younger participants (aged 22–48 years) by swpPERG using the EvokeDx^®^ system. This was consistent with the fact that the number of retinal cells, such as photoreceptor cells^16–19^ and ganglion cells^20^, decline with age. Additionally, age-related retinal changes regarding macular pigment levels as measured by macular pigment optical density have been reported^21^.

Positive correlations between SNR and the macular RNFL and GCL volumes were shown in participants with SNR ≥ 1. Also, participants who showed SNR ≥ 1, and did respond to the smallest stimulus, had significantly greater RNFL volume. Because the swpPERG data were obtained under photopic condition, the responses most likely reflect the function of macular area. Pattern ERG is generally supposed to reflect at least ganglion and inner retinal function^22^ as shown in animal experiments in which chemical suppression of synaptic transmission showed reduction in responses^23^. However, whether swpPERG can assess the condition of the inner retina remains to be clarified. In the current study, we found that function was related to the volume of neural tissue, which supports this idea. The impact of SNR on representing inner retinal function should be studied in the future through measurement in patients with inner retinal degenerative diseases.

The swpPERG measurements have distinct advantages such as using the EvokeDx^®^ system do not require dilating the pupils, wearing contact lenses for attaching electrodes, and performing after dark adaptation and in a dark room. The time to assess is also relatively short. This measurement could also be valuable in the field of paediatric ophthalmology, which has considerable practical implications when dealing with young patients.

This study has some limitations. We had a relatively small sample size, participants were relatively young, and we did not evaluate subclinical cataract, if any. Nonetheless, there were variations in visual function among individuals and the EvokeDx^®^ system detected them.

The SNR as determined on ERG recorded using the EvokeDx^®^ system were correlated with age and macular volume in healthy adults. The system detected slight differences in visual function related to age and/or retinal morphological variations. Future study incorporating the system will help early diagnosis and neuroprotective studies that require the detection of slight differences in visual function.

## Methods

### Participants

Thirty-five left eyes were evaluated in 35 healthy adult volunteers (18 men and 17 women) between July 2016 to February 2019 at the Medical Retina Division, Department of Ophthalmology, Keio University Hospital. The data of left eyes were analysed. No participant had ocular disease, except for refractive error. The best-corrected visual acuity (BCVA) of all participants was 1.2 in decimal (−0.079 in LogMAR). Participants with high myopia (>6 diopters) were excluded to avoid including pathological myopia.

The study adhered to the tenets of the Declaration of Helsinki and approved by the Ethics Committee of Keio University School of Medicine (Tokyo, Japan; 20150116). The study was registered as UMIN000017845. Written informed consent was obtained from all participants.

### ERG

The swpPERG was recorded by EvokeDx^®^ (Konan Medical, California, USA) system. Data were obtained using spatial-patterned (horizontal grating) and contrast-reversed stimuli of size 1 (thickest) to 6 using skin electrodes under photopic condition according to the manufacturer’s protocol. The distance from the participants to the monitor was 65 cm, and the monitor size was 17 inches. Participants used contact lenses during the examination, if they needed corrections to obtain BCVA. The frequency component after stimulation by each size of stimulus was compared with background brain activity recorded as noise, and the data were expressed by SNR. Measurements were performed twice, and the greater value was included in the analyses.

### OCT

Three-dimensional OCT images were obtained using spectral domain OCT (Heidelberg Spectralis OCT, Heidelberg Engineering GmbH, Dossenheim, Germany). The macular volume in each layer was evaluated using the built-in software of the OCT device.

### Statistical analyses

Data are shown in mean ± standard deviation. McNemar test, Two-tailed t-test, and Pearson correlation coefficient (IBM SPSS Statistics version 24.0) were used to analyse the data. *P < *0.05 was considered to be statistically significant.

### Approval, Accordance and Informed Consent Statements

The study adhered to the tenets of the Declaration of Helsinki and approved by the Ethics Committee of Keio University School of Medicine (Tokyo, Japan; 20150116). The study was registered as UMIN000017845. Written informed consent was obtained from all participants.

## Supplementary information

Dataset 1

## Data Availability

The protocol and the datasets generated during and/or analyzed during the current study are available from the corresponding author upon reasonable request.
