# Supplementary material for: Spatial-sweep steady-state pattern electroretinography can detect subtle differences in visual function among healthy adults
Source: Sci Rep. 2019 Dec 2;9:18119. doi: 10.1038/s41598-019-54606-z (PMC6889279; doi:10.1038/s41598-019-54606-z)
Supplement: Supplementary file 1 — Dataset 1 [file 41598_2019_54606_MOESM1_ESM.pdf]

# Spatial-sweep steady-state pattern electroretinography can detect subtle differences in visual function among healthy adults

Sakiko Minami[1,2,5], Norihiro Nagai[1,3], Misa Suzuki[1,4], Toshihide Kurihara[1], Hideki Sonobe[1], Kazuhiro Watanabe[1], Hajime Shinoda[1], Hitoshi Takagi[5], Kazuo Tsubota[1], Yoko Ozawa[1,3].

1 Department of Ophthalmology, Keio University School of Medicine, 2 Department of Ophthalmology, Inagi Municipal Hospital, 3 Laboratory of Retinal Cell Biology, Department of Ophthalmology, Keio University, School of Medicine 4 Department of Ophthalmology, Yokohama City University, 5 Department of Ophthalmology, St. Marianna University School of Medicine.

Running head: Variation of visual function among healthy adults

***Corresponding author:**
**Yoko Ozawa, M.D., Ph.D.**
Laboratory of Retinal Cell Biology
Department of Ophthalmology
Keio University School of Medicine
35 Shinanomachi, Shinjuku-ku, Tokyo 160-8582, Japan
Phone: +81-3-5363-3869, Fax: +81-3-5363-3869
E-mail: ozawa@a5.keio.jp

**Supplementary table 1. Correlation between SNR and Age at Each Stimulus Size among Participants Who Showed SNR ≥1**

| | r | *P* |
|---|---|---|
| **Stimulus size 1** | -0.234 | 0.214 |
| **Stimulus size 2** | -0.288 | 0.137 |
| **Stimulus size 3** | -0.308 | 0.097 |
| **Stimulus size 4** | -0.312 | 0.093 |
| **Stimulus size 5** | -0.500 | 0.029* |
| **Stimulus size 6** | 0.279 | 0.721 |

Pearson correlation coefficient. SNR, signal to noise ratio. *$P<0.05$.

**Supplementary table 2. Mean Macular Volume of All Participants**

| Macular Volume ($mm^3$) | Range | ( | Mean | ± | SD | ) |
|---|---|---|---|---|---|---|
| Total | 7.92 to 9.30 | ( | 8.53 | ± | 0.36 | ) |
| RNFL | 0.77 to 1.38 | ( | 0.99 | ± | 0.13 | ) |
| GCL | 0.83 to 1.19 | ( | 1.05 | ± | 0.09 | ) |
| IPL | 0.74 to 0.97 | ( | 0.86 | ± | 0.07 | ) |
| INL | 0.85 to 1.12 | ( | 0.96 | ± | 0.07 | ) |
| OPL | 0.69 to 1.04 | ( | 0.84 | ± | 0.10 | ) |
| ONL | 1.31 to 2.00 | ( | 1.63 | ± | 0.16 | ) |
| ELM-RPE | 2.15 to 2.37 | ( | 2.27 | ± | 0.05 | ) |

RNFL, retinal nerve fibre layer; GCL. ganglion cell layer; IPL, inner plexiform layer; INL, inner nuclear layer; OPL, outer plexiform layer; ONL, outer nuclear layer; ELM, external limiting membrane; RPE, retina pigment epithelium.

**Supplementary table 3. Correlation between SNR and Retinal Nerve Fibre Layer and Ganglion Cell Layer Volumes among Participants Who Showed SNR ≥ 1**

| | | RNFL Total volume | | GCL Total volume | |
|---|---|---|---|---|---|
| | **n** | **R** | ***P*** | **R** | ***P*** |
| **Stimulus size 1** | 31 | -0.162 | 0.384 | -0.032 | 0.863 |
| **Stimulus size 2** | 28 | 0.049 | 0.805 | 0.046 | 0.816 |
| **Stimulus size 3** | 30 | 0.315 | 0.090 | 0.264 | 0.158 |
| **Stimulus size 4** | 30 | 0.409 | 0.025* | 0.039 | 0.838 |
| **Stimulus size 5** | 19 | 0.326 | 0.173 | 0.567 | 0.011* |
| **Stimulus size 6** | 4 | -0.154 | 0.846 | -0.003 | 0.997 |

Pearson's product-moment correlation. SNR, signal to noise ratio; RNFL, retinal nerve fibre layer; GCL, ganglion cell layer. *$P<0.05$.
